# 

**Published:** 1903-09-19

**Authors:** 


NOTICE TO CORRESPONDENTS.

All MSS., Letters, Books for Review, and other matters intended for the Editor should be addressed The Editor, "The Hospital," The Hospital Buildings, 28 & 29 Southampton Street, Strand, W.C.

The Editor cannot undertake to return rejected MS., even when accompanied by stamped directed envelope.

**Notice to Advertisers and Subscribers.**

All Advertisements, Orders for Copies of The Hospital, and Business Communications should be addressed to **THE MANAGER, (Not to the Editor)**, The Hospital, 28 & 29 Southampton Street, Strand, London, W.C.

The Cost of Subscription, post free, is as follows.—Per week, 3½d.; per quarter, 3s. 9d.; per half-year, 7s. 6d.; per annum, 15s. Foreign and Colonial:—Per annum, 19s. 6d., payable, in all cases, in advance.

**NOTICE.—Vol. XXXIII. of "The Hospital" (October 1902 to March 1903) in handsome cloth binding, will shortly be on sale at the office of this Journal, price 7s. 6d. Cases for binding the Numbers contained in this Volume 1s. 6d. Index to Vol. XXXIII., 2½d., post free.**

**The monthly part for September is now ready. Price 1s., by post 1s. 3d.**

### THE STUDENT OF MEDICINE.

### APPOINTMENTS.

**Marmaduke Bannister**, M.B.Lond., M.R.C.S.Eng., Honorary Physician of the Blackburn and East Lancashire Infirmary. **G. A. Bosson**, M.R.C.S., L.R.C.P., District Medical Officer of the Lewisham Union. **R. T. Bruce**, M.B., M.S.Edin., District and Workhouse Medical Officer of the Thame Union. **F. J. Butt**, M.B., C.M., reappointed Medical Officer of Health to the Hoole Urban Council. **J. P. Byrne**, L.R.C.S.I., House Surgeon, Provincial Hospital, Port Elizabeth, South Africa. **Alfred Campbell**, F.R.C.S.Edin., M.R.C.S.Eng., Assistant Medical Superintendent of the Hospital for the Insane at Toowoomba, Queensland. **T. F. Clemson**, M.R.C.S., L.R.C.P., Certifying Factory Surgeon for the Wootton Bassett District, Wiltshire. **D. Connor**, M.R.C.S., District Medical Officer and Public Vaccinator, Victoria Plains, West Australia. **John Benson Cooke**, L.R.C.P., L.R.C.S.Edin., Medical Officer to H.M. Convict Prison, Portland. **Archibald Cuff**, M.B., B.C.Cantab., Honorary Surgeon to the Sheffield Royal Infirmary. **A. R. F. Exham**, M.D., B.Ch. Dub., District Medical Officer of the Drayton Union. **W. L. Garner**, M.B., B C.Camb., Medical Officer of the Ampthill Union Workhouse. **Christina L. Goode**, M.B., Assistant Medical Officer, West Ham Infirmary, Leytonstone, N.E. **J. Sangster Greig**, M.B.Aberd., Medical Officer, West Ham Union Workhouse, Leytonstone, N.E. **H. D. Haworth**, M.B., Ch.B., House Physician to the Manchester Royal Infirmary. **J. F. Hodgson**, M.D., Ch.B.Vict., Assistant Medical Officer of Health of the County Borough of Halifax. **H. G. Holmes**, M.B., M.Ch Syd., Government Medical Officer and Vaccinator at Warialda, N.S.W. **T. Howard**, M.B., B.S.Irel., District Medical Officer of the Weymouth Union. **J. A. Hunt**, L.R.C.P.Edin., M.R.C.S.Eng., Certifying Factory Surgeon for the Borrowash District, Derbyshire. **A. E. Johnson**, M.B., Ch.B.,

House Surgeon to the Manchester Royal Infirmary. **Percy Kitchin**, M.R.C.S., L.R.C.P., Certifying Factory Surgeon for the Newport District, County Salop. **J. P. Lavery**, L R.C.P.&S.Edin., L.F.P.S.Glasg., Certifying Factory Surgeon for the Dundalk District, County Louth. **D. R. Macdonald**, L.R.C.P., L.R.C.S.Edin., Certifying Factory Surgeon for the Dunbar District, Haddington. **R. B. Macpherson**, M.D.Glasg, Certifying Factory Surgeon for the Cambuslang District, co. Lanark. **R. H. Martin**, M.B., B.S.Edin., Second Assistant Medical Officer to the Central London Sick Asylum District. **T. W. Ogilvie**, M.B., M.S., District Medical Officer of the East Retford Union. **C. S. O'Neill**, M.B., Ch.B., House Physician to the Manchester Royal Infirmary. **A. Osborne**, M.R.C.S., L.R.C.P., District Medical Officer of the Barnstaple Union. **H. J. Penny**, L.K.Q.C.P.I., Public Vaccinator for the Portland District, Tasmania. **J. Reidy**. L.R.C.P.&S.Edin., District Medical Officer of St. George-in-the-East Parish. **J. M. Rhodes**, M.D.Brux., L.R.C P., L.R.C S.Edin., Certifying Factory Surgeon for the East Salford District, Lancaster. **J. W. Sandoe**, M.D.Durh., B.S, Certifying Factory Surgeon for the Silverton District, County Devon. **J. G. V. Sapp**, M.B., C.M.Edin. D.P.H., District Medical Officer of the Strand Union. **Joseph Scott**, M.B., C.M.Glasg, Medical Superintendent, Government Indo-European Telegraphs, Teheran. Persia. **Robert G. Scott**, M.B., C.M., Honorary Medical Officer to the Hobart Hospital, Tasmania. **W. T. Scott**, M.B., B.C.Camb., House Physician to the Royal Berks Hospital, Reading. **W. B. Secretan**. M.B.Lond., F.R.C.S., House Surgeon to the Royal Berks Hospital, Reading. **W. F. Shaw**, M.B., Ch.B., House Surgeon to the Manchester Royal Infirmary. **James G. Sheahan**, L.R.C.P.&S.Edin., L.F.P.S.Glasg., Physician to Out-patients, St. Vincent's Hospital, Melbourne. **D. Ap. Simon**, M.B., Ch.B.Glasg., District Medical Officer of the Llandilofawr Union. **W. G. Stewart**, M.B., B.S.Lond., District Medical Officer of the Ware Union. **Robert B. Stoney**, M.B., Ch.B.Dub., Medical Officer for the Aboriginal Station at Cumeroogunga, N.S.W. **F. W. Taylor**, M.B., C.M., Honorary Assistant Physician of the Blackburn and East Lancashire Infirmary. **James Thompson**, M.B., B.S.Melb., Resident Medical Officer to the Perth Public Hospital, West Australia. **T. A. Walker**, L.R.C.P.Edin., M.R.C.S., District Medical Officer of the Weymouth Union. **T. D. White**, L.S.A., Certifying Factory Surgeon for the Southminster District, Essex. **G. C. Winchester**, M.B., M.S.Edin., District Medical Officer of the Eastry Union. **J. Wright-Grant**, M.B., C.M., Physician to the Daneswood Sanatorium, Woburn Sands.

### "The Hospital" Institutional Library.

"Hospitals and Asylums of the World." 4 Vols., with a Portfolio of Plans, complete £12 12s.

"Burdett's Hospitals and Charities, 1903." 5s. net.

"Burdett's Official Nursing Directory, 1903." 3s. net.

"Cottage Hospitals, General, Fever, and Convalescent." 10s. 6d.

"Uniform System of Accounts for Hospitals and Public Institutions." New Edition. 4s. net.

"Hospital Expenditure: The Commissariat." 2s. 6d.

"A Legal Handbook for the Use of Hospital Authorities." 2s. 6d.

"The Cottage Hospital Case Book or Register of Patients." 10s. and 12s. 6d.

All these are published by the Scientific Press, Ltd., and may be obtained through any bookseller, or direct from the publishers. 28 and 29 Southampton Street, Strand, London, W.C.

### Special Text-Books for Nurses.

### OPHTHALMIC NURSING.

By SYDNEY STEPHENSON, F.R.C.S.E.

Price **3/6** net, **3/10** post free.

A valuable guide on the Nursing of Eye Cases.

### NURSING IN DISEASES OF THE THROAT, NOSE, AND EAR.

By P. MACLEOD YEARSLEY, F.R.C.S.

Price **2/6.**

Contains useful hints on these special subjects.

### THE ART OF MASSAGE.

By A. CREIGHTON HALE.

Price **6/-.**

Every Nurse must find a knowledge of Massage useful and interesting.

### FEVERS AND INFECTIOUS DISEASES.

By Dr. WILLIAM HARDING.

Price **1/-.**

An excellent little handbook.

### NOTES ON PHARMACY AND DISPENSING.

By C. J. S. THOMPSON.

Price **1/-.**

An admirable introduction to Dispensing.

### MENTAL NURSING.

By Dr. WILLIAM HARDING.

Price **1/-.**

Full of useful advice to Nurses in Mental Cases.

### OUTLINES OF INSANITY.

By Dr. WALMSLEY.

A Treatise on the Salient Features of Insanity. Very useful to the intelligent Mental Nurse.

*Obtainable of all Booksellers, and published by*
THE SCIENTIFIC PRESS, LIMITED, 28 & 29 Southampton Street, Strand, London,
*who will send their latest Catalogue of Nursing Text-books post free to any Nurse on application.*

